# TMED3 promotes the development of malignant melanoma by targeting CDCA8 and regulating PI3K/Akt pathway

**DOI:** 10.1186/s13578-023-01006-6

**Published:** 2023-03-29

**Authors:** Xianling Guo, Xiaolan Yin, Yu Xu, Liang Li, Min Yuan, Huaxin Zhao, Yuxiong Jiang, Xiujuan Shi, Hongda Bi, Yeqiang Liu, Yong Chen, Qing Xu

**Affiliations:** 1 Department of Oncology, Dermatology Hospital, Tongji University, Shanghai, 200092 China; 2 Department of Oncology, Shanghai Tenth People’s Hospital, Tongji University, Shanghai, 200092 China; 3 Tongji University Cancer Center, Shanghai, 200072 China; 4 Department of Musculoskeletal Oncology, Fudan University Shanghai Cancer Center, Shanghai, 200032 China; 5 Department of Oncology, Shanghai Medical College, Fudan University, Shanghai, 200032 China; 6 Department of Dermatologic Surgery, Dermatology Hospital, Tongji University, Shanghai, 200092 China; 7 Tongji University School of Medicine, Tongji University, Shanghai, 200092 China; 8 Department of Plastic Surgery Changhai Hospital, 168# Changhai Road, Shanghai, 200433 China; 9 Department of Pathology, Dermatology Hospital, Tongji University, Shanghai, 200092 China

**Keywords:** Malignant melanoma, TMED3, CDCA8, PI3K/Akt pathway

## Abstract

**Background:**

Transmembrane emp24 domain containing (TMED) proteins are known to play pivotal roles in normal development, but have been reported to be implicated in pancreatic disease, immune system disorders, and cancers. As far as TMED3 is concerned, its roles in cancers are controversial. However, evidence describing TMED3 in the context of malignant melanoma (MM) is scarce.

**Results:**

In this study, we characterized the functional significance of TMED3 in MM and identified TMED3 as a tumor-promoting factor in MM development. Depletion of TMED3 arrested the development of MM in vitro and in vivo. Mechanistically, we found that TMED3 could interact with Cell division cycle associated 8 (CDCA8). Knocking down CDCA8 suppressed cell events associated with MM development. On the contrary, elevating CDCA8 augmented cell viability and motility and even reversed the inhibitory effects of TMED3 knockdown on MM development. On the other hand, we found that the levels of P-Akt and P-PI3K were decreased in response to TMED3 downregulation, which was partially abolished following SC79 treatment. Thus, our suspicion was that TMED3 exacerbates MM progression via PI3K/Akt pathway. More notably, previously decreased P-Akt and P-PI3K in TMED3-depleted cells were rescued after overexpressing CDCA8. Also, previously impaired cell events due to CDCA8 depletion were ameliorated after SC79 addition, implying that TMED3 regulates PI3K-AKT pathway via CDCA8, thereby promoting MM development.

**Conclusions:**

Collectively, this study established the link between TMED3 and MM, and provides a potential therapeutic intervention for patients with MM harboring abundant TMED3.

**Supplementary Information:**

The online version contains supplementary material available at 10.1186/s13578-023-01006-6.

## Background

Malignant melanoma (MM) is the fastest growing cancer in whites worldwide [1, 2]. The increase in its incidence results from increased sun exposure due to changes in clothing habits and lifestyles over the past few decades. Most melanomas appear in pre-existing moles in areas that are exposed to sunlight. Once undetected and cleared early, it may spread rapidly through the vascular system [3]. Till now, the treatment outcomes for the disease have been still dismal, with most patients dying from refractory brain metastases [1, 4].

Transmembrane emp24 domain containing (TMED) proteins constitute a highly conserved protein family [5, 6]. Members of this family share a similar domain structure, with a coiled-coil domain, a transmembrane domain, and a short cytoplasmic tail with several highly conserved motifs [7]. TMED proteins dimerize and bind to the coatomer protein complexes to moderate the transport of various cargo proteins, such as glycosylphosphatidylinositol-anchored proteins, G-protein-coupled receptors, and Wnt ligands [8–11]. On the other hand, TMED proteins are engaged in normal development, but have been evidenced to be involved in pancreatic disease, immune system disorders, and cancers [7, 8]. Specifically, in ovarian cancer, TMED2 promotes cell proliferation, migration, and invasion by activating the IGF2/IGF1R/PI3K/Akt pathway [12]. Besides, TMED9 is revealed to be strongly expressed in various human malignancies including breast cancer, colon cancer, ovarian cancer, gastrointestinal cancer, lung cancer and hepatocellular cancer [13]. TMED10 induces autophagy in papillary thyroid cancer cells by activating the AMPK/mTOR pathway [14]. As far as TMED3 is concerned, its functional roles in cancers are controversial. TMED3 suppresses distant colon cancer metastases [9], but promotes tumor progression in liver cancer and breast cancer [15, 16]. However, evidence describing TMED3 in the context of MM is scarce.

The aim of this study was to determine the functional importance and action’s mechanism of TMED3 in MM. To this end, TMED3 levels were investigated via IHC analysis and qPCR detection. Besides, we used shRNA expressing TMED3 to knock down TMED3 in A375 and OM431 cells. Moreover, we performed cell and animal experiments to verify the effects of TMED3 depletion on the development of MM. More importantly, we made a preliminary exploration on the mechanism by which TMED3 regulates MM. Therefore, we identified TMED3 as a tumor promotor in MM, which may be a promising therapeutic target for MM.

## Results

### TMED3 expression is abundant in malignant melanoma

**Fig. 1:**
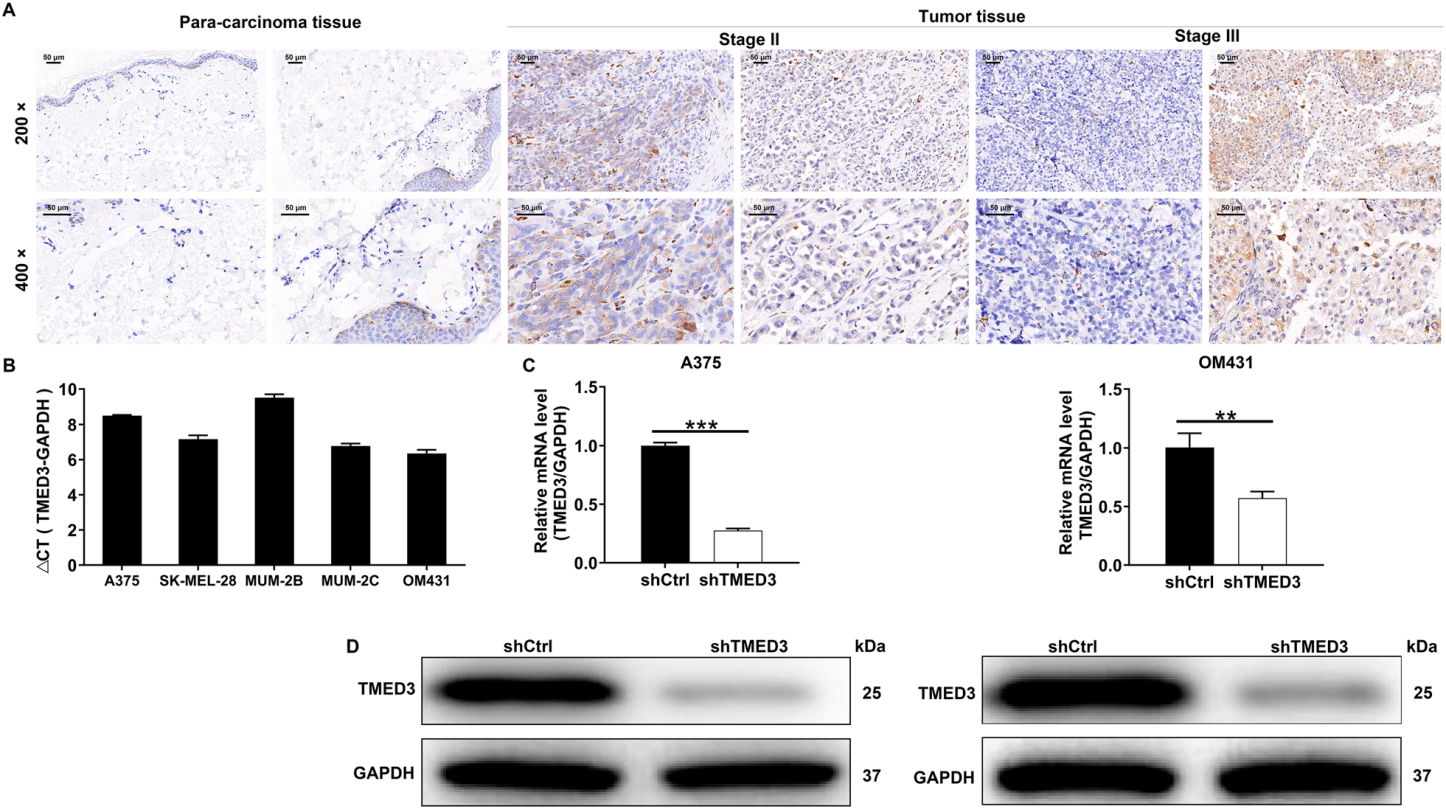
TMED3 was abundantly expressed in malignant melanoma. **A** The protein expression of TMED3 in melanoma and normal tissues was detected by IHC. **B** The mRNA expression of TMED3 in melanoma cell lines was detected by qRT-PCR. **C** The TMED3 expression in A375 and OM431 cell lines after infection was analyzed by qRT-PCR. **D** The expression of TMED3 protein in A375 and OM431 cell lines after infection was detected by western blot. ** *P* < 0.01, *** *P* < 0.001

To clarify the underlying roles of TMED3 in MM progression, we performed IHC analysis on a tissue microarray containing 167 melanoma specimens and 30 non-tumor samples. The results suggested that TMED3 protein expression was significantly upregulated in 52.1% of melanoma specimens (87/167), while the majority of 30 non-tumor samples exhibited low TMED3 levels (29/30) (Fig. 1A and Table 1). We next detected TMED3 mRNA levels in a panel of melanoma cell lines. As expected, all melanoma cells harbored high TMED3 mRNA levels (Fig. 1B). To investigate the clinical significance of TMED3 in MM, we analyzed the relationship between the expression patterns of TMED3 and the pathological parameters of patients, and observed that high TMED3 expression was associated with more distant lymphatic metastasis (*P* = 0.014) and higher pathological stage (*P* = 0.012) (Table 2), which was also verified by Spearman rank correlation analysis (Table 3). Collectively, these data suggested that TMED3 might be a tumor promotor in MM outgrowth.

**Table 1:** Expression patterns of TMED3 in malignant melanoma tissues and para-carcinoma tissues revealed in immunohistochemistry analysis

TMED3 expression	Tumor tissue		Para-carcinoma tissue		*P* value
	Cases	Percentage	Cases	Percentage	
Low	80	47.9%	29	96.7%	*P* < 0.001
High	87	52.1%	1	3.3%

**Table 2:** Relationship between TMED3 expression and tumor characteristics in patients with malignant melanoma

Features	No. of cases	TMED3 expression		*P* value
		low	high	
All cases	167	80	87	
Age (years)				0.931
≤ 5	85	41	44	
> 55	82	39	43	
Gender				0.972
Male	90	43	47	
Female	77	37	40	
Tumor infiltrate				0.343
T2	6	3	3	
T3	26	9	17	
T4	74	36	38	
lymphatic metastasis (N)				0.014
N0	94	46	48	
N1	10	2	8	
N2	4	0	4	
Stage				0.012
I	6	3	3	
II	84	43	41	
III	12	2	10	
IV	4	0	4

**Table 3:** Relationship between TMED3 expression with lymphatic metastasis and pathological stage in patients with malignant melanoma

		TMED3
lymphatic metastasis (N)	Spearman correlation	0.238
	Signification (double-tailed)	0.013
	N	108
Stage	Spearman correlation	0.244
	Signification (double-tailed)	0.012
	N	106

### TMED3 promotes malignant melanoma cell proliferation and metastasis in vitro

**Fig. 2:**
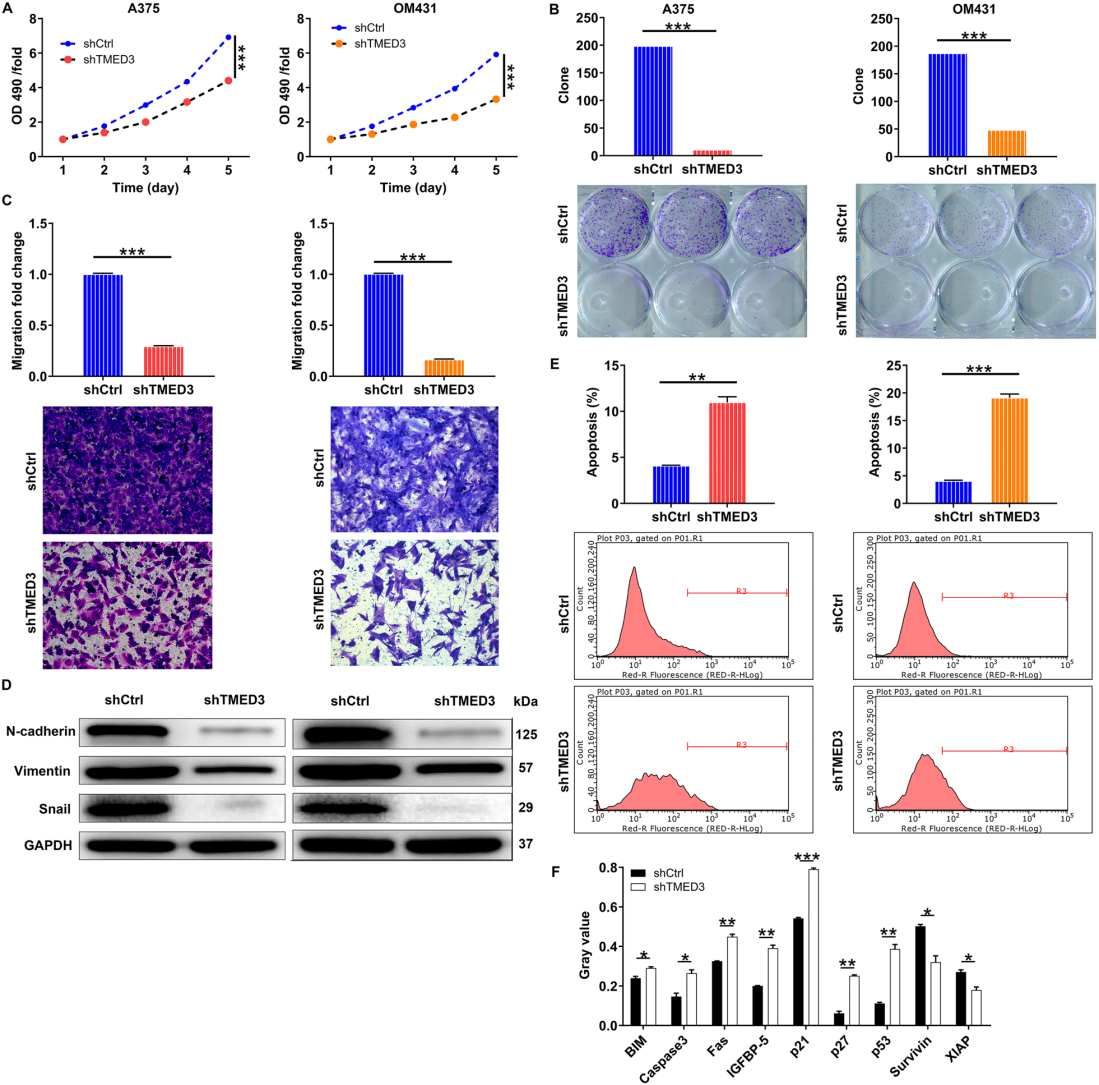
TMED3 knockdown inhibited cell proliferation, colony formation and migration, induced cell apoptosis. **A** MTT assay was used to detect the effects of TMED3 knockdown on cell proliferation of A375 and OM431 cells. **B** The abilities of A375 and OM431 cells to form colony after infection were assessed. **C** The effects of TMED3 knockdown on A375 and OM431 cell migration capacities were detected by transwell assay. **D** The western blot was employed to detect the levels of EMT-associated elements in A375 and OM431 cells upon silencing TMED3. **E** Flow cytometry was performed to evaluate the effects of TMED3 knockdown on cell apoptosis of A375 and OM431 cells. **F** The changes in apoptosis-related proteins were analyzed in A375 cells following infection by a Human Apoptosis Antibody Array. Protein level was presented in gray value. The data were expressed as mean ± SD. * *P* < 0.05, ** *P* < 0.01, *** *P* < 0.001

To explore the biological functions of TMED3 in MM in vitro, we first knocked down TMED3 in A375 and OM431 cells, further examined TMED3 expression and found that TMED3 was markedly downregulated at both mRNA and protein levels (Fig. 1C, D). Thus, these both cell models were employed for the following cell experiments. We found that in TMED3-deficient A375 and OM431 cells, the capacities of cell proliferation were limited (Fig. 2A). Besides, we observed that the abilities of both cells to form colony were also suppressed (Fig. 2B). Additionally, we found that silencing TMED3 in A375 and OM431 cells significantly inhibited cell migration, as measured by the transwell assay (Fig. 2C). To verify that the changes in migration were not caused by the inhibition of proliferation, we further tested the levels of EMT-related proteins in TMED3-insufficient A375 and OM431 cells, suggesting the downregulation of N-cadherin and Snail while the upregulation of Vimentin (Fig. 2D). Furthermore, silencing TMED3 caused cell apoptosis enhanced (Fig. 2E), which might be induced in a manner of increasing BIM, Caspase3, Fas, IGFBP-5, p21, p27 and p53 while decreasing Survivin and XIAP (Fig. 2F). Taken together, TMED3 could promote MM cell proliferation and metastasis while suppress apoptosis in vitro*.*

### TMED3 depletion impairs the growth of xenograft tumors

**Fig. 3:**
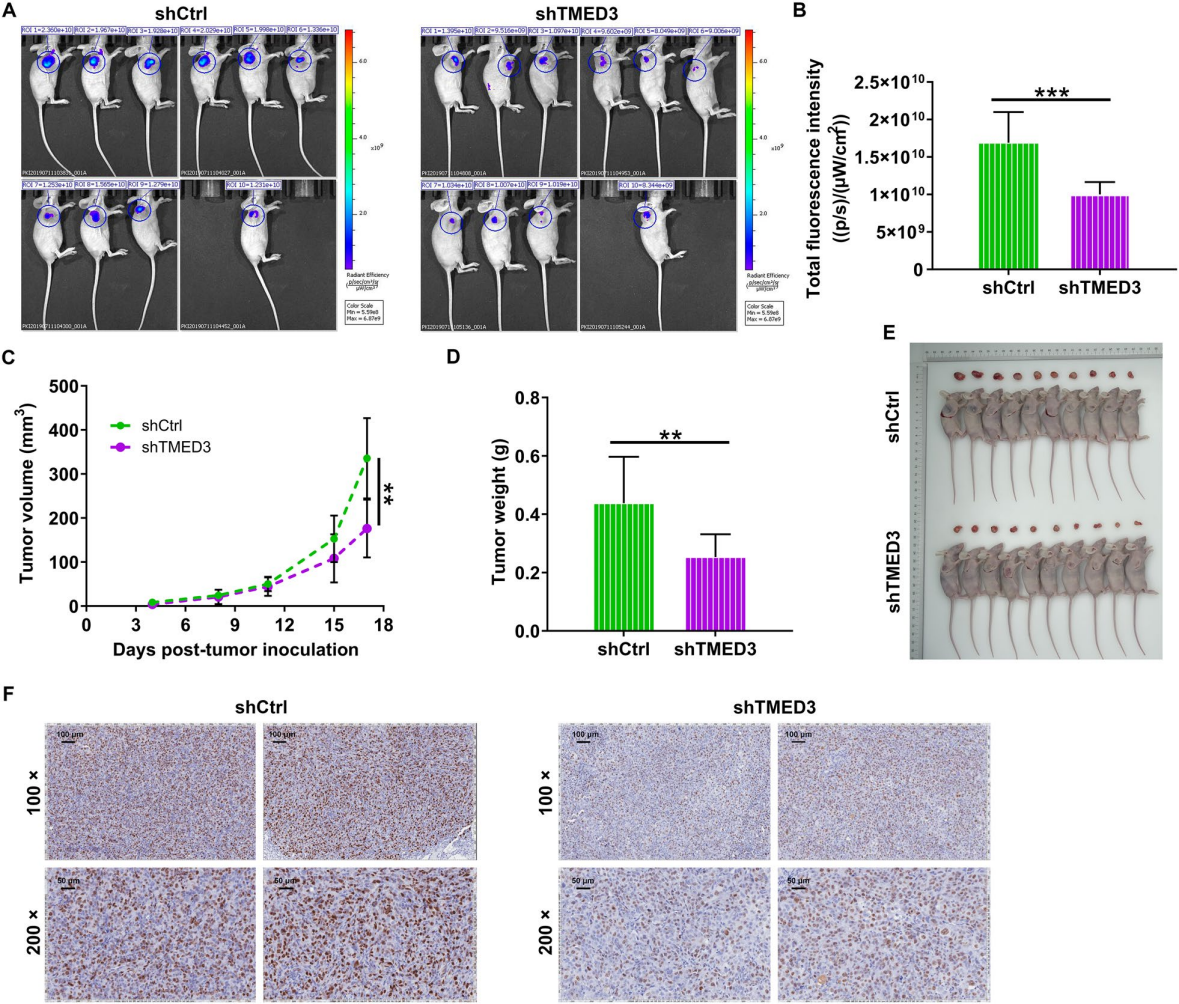
TMED3 knockdown inhibited tumor growth of malignant melanoma in vivo. **A** A nude mice model of TMED3 knockdown was constructed via subcutaneous injection of A375 cells. **B**–**D** The fluorescence (**B**), volume (**C**) and weight (**D**) of xenograft tumors in shCtrl and shTMED3 groups were measured in indicated time. **E** The photos of tumors removed from mice models were collected. **F** The pattern of Ki-67 was detected by IHC analysis in tumor sections from shCtrl and shTMED3 mice models. The data were expressed as mean ± SD. ** *P* < 0.01, *** *P* < 0.001

In this section, we wondered whether TMED3 depletion could influence melanoma tumor outgrowth. Thus, we subcutaneously injected TMED3-deficient A375 cells into nude mice (Fig. 3A), and measured tumor growth indicators. The results from the detection of fluorescence showed the fluorescence intensity in TMED3-deficient A375 cell-xenografted tumors was diminished (Fig. 3B). More strikingly, depletion of TMED3 evidently arrested tumor growth, mainly resulting in a restraint of tumor volume and weight (Fig. 3C–E). We further examined the expression of Ki-67, a proliferation index, which was found to be decreased in tissues from nude mice derived from TMED3-deficient cells (Fig. 3F). Together, these data confirmed that the inhibition of TMED3 could significantly delay the growth of xenografted tumors.

### Exploration of the downstream mechanism of TMED3 regulating malignant melanoma

**Fig. 4:**
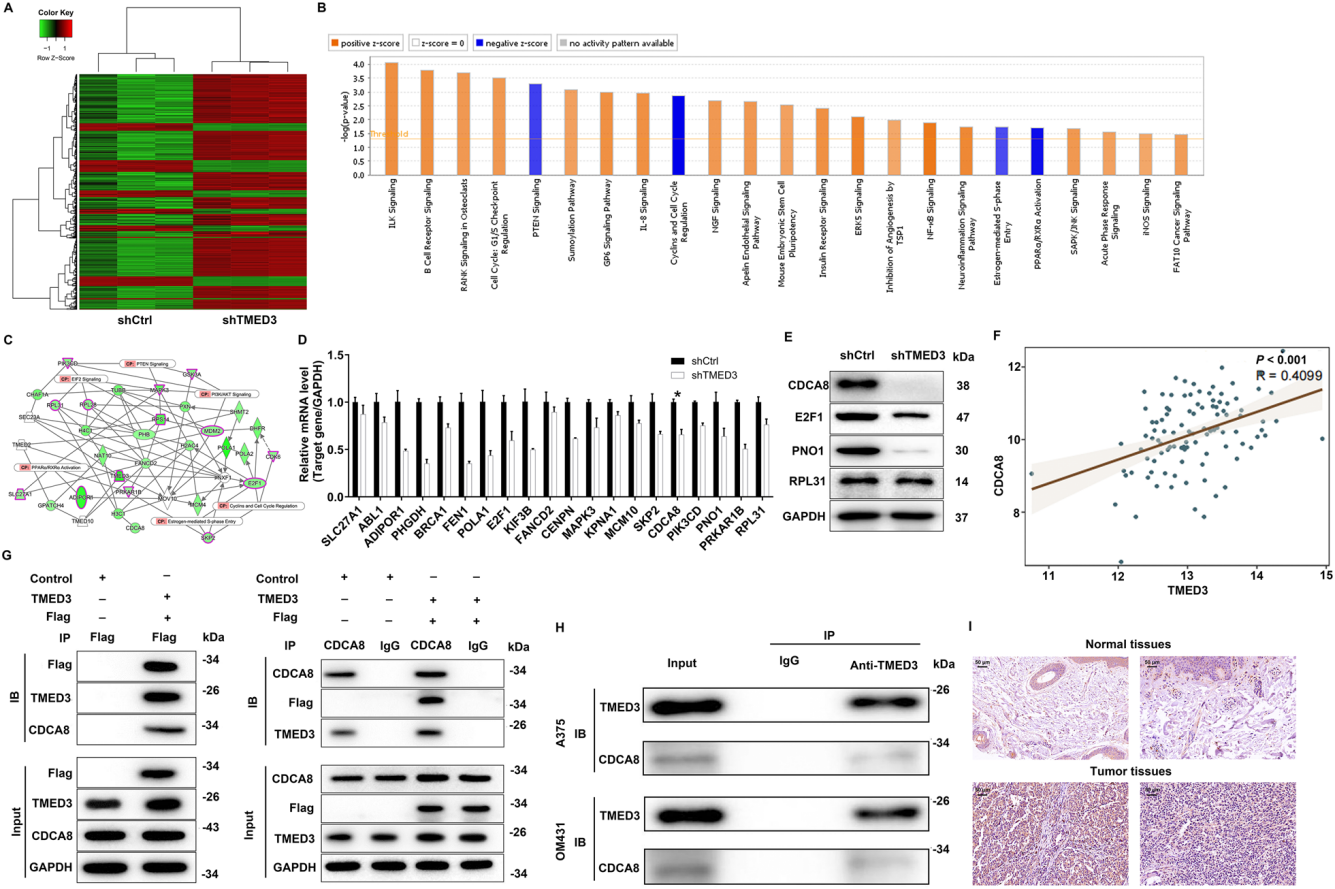
CDCA8 was identified as the downstream target of TMED3 regulating malignant melanoma. **A** PrimeView human gene expression array (3 v 3) was performed to identify differentially expressed genes in A375 cells with or without TMED3 knockdown. **B** The enrichment of the DEGs in canonical signaling pathways was analyzed by IPA. **C** The IPA analysis was performed to produce the TMED3-related interaction network. **D**, **E** The expression of several most significantly down-regulated DEGs was further determined via qRT-PCR (**D**) and western blotting (**E**). **F** The correlation between TMED3 and CDCA8 was investigated based on the samples from TCGA-SKCM. **G**, **H** Exogenous (**G**) and endogenous (**H**) Co-IP assay was used to verify whether there was protein interaction between TMED3 and CDCA8. **I** The protein levels of CDCA8 in melanoma and normal tissues were detected via IHC staining

Having shown that TMED3 inhibition displayed potent anti-melanoma in vitro and in vivo, we further investigated the underlying mechanism of TMED3’s action in melanoma. Thus, a GeneChip primeview human PathArray^™^ was performed in TMED3-deficient A375 cells, where we obtained 708 up-regulated and 190 down-regulated genes in comparison with control shCtrl cells (Fig. 4A). Subsequently, the enrichment of these differential expressed genes (DEGs) in the classic pathways resulted that Cyclins and Cell Cycle Regulation, PPARα/RXRα Activation, PTEN Signaling, and Estrogen-mediated S-phase Entry pathways were significantly inhibited after silencing TMED3 (Fig. 4B). In addition, a network between TMED3 with the above pathways was established, indicating that TMED3 could affect GSK3A, MAPK3, MDM2, PIK3CD, ADIPOR1, PRKAR1B, SLC27A1, RPL28, RPL31, RPS14, E2F1, SKP2 and CDK6 (Fig. 4C). We further detected the mRNA levels of these genes in TMED3-deficient A375 cells, 4 of which were used for western blot verification. The data demonstrated that CDCA8 mRNA and protein levels were obviously downregulated (Fig. 4D, E), implying CDCA8 as a downstream gene of TMED3. Moreover, the correlation between TMED3 and CDCA8 was investigated through analyzing RNA-seq readcounts expression data of MM samples from TCGA database. We found that there was a positive correlation between the both with statistical significance (Fig. 4F). More intriguingly, the exogenous Co-IP experiment showed that TMED3 interacted with CDCA8 (Fig. 4G). Also, endogenous TMED3 was also present in endogenous CDCA8 immunoprecipitates from A375 and OM431 cells (Fig. 4H). Beyond that, CDCA8 expressed at a high level in MM tissues relative to non-tumor tissues (Fig. 4I). Based on the above, we identified that CDCA8 was the downstream target of TMED3-mediated MM.

### TMED3 accelerates malignant melanoma development via the regulation of CDCA8 and PI3K/AKT signaling pathway

**Fig. 5:**
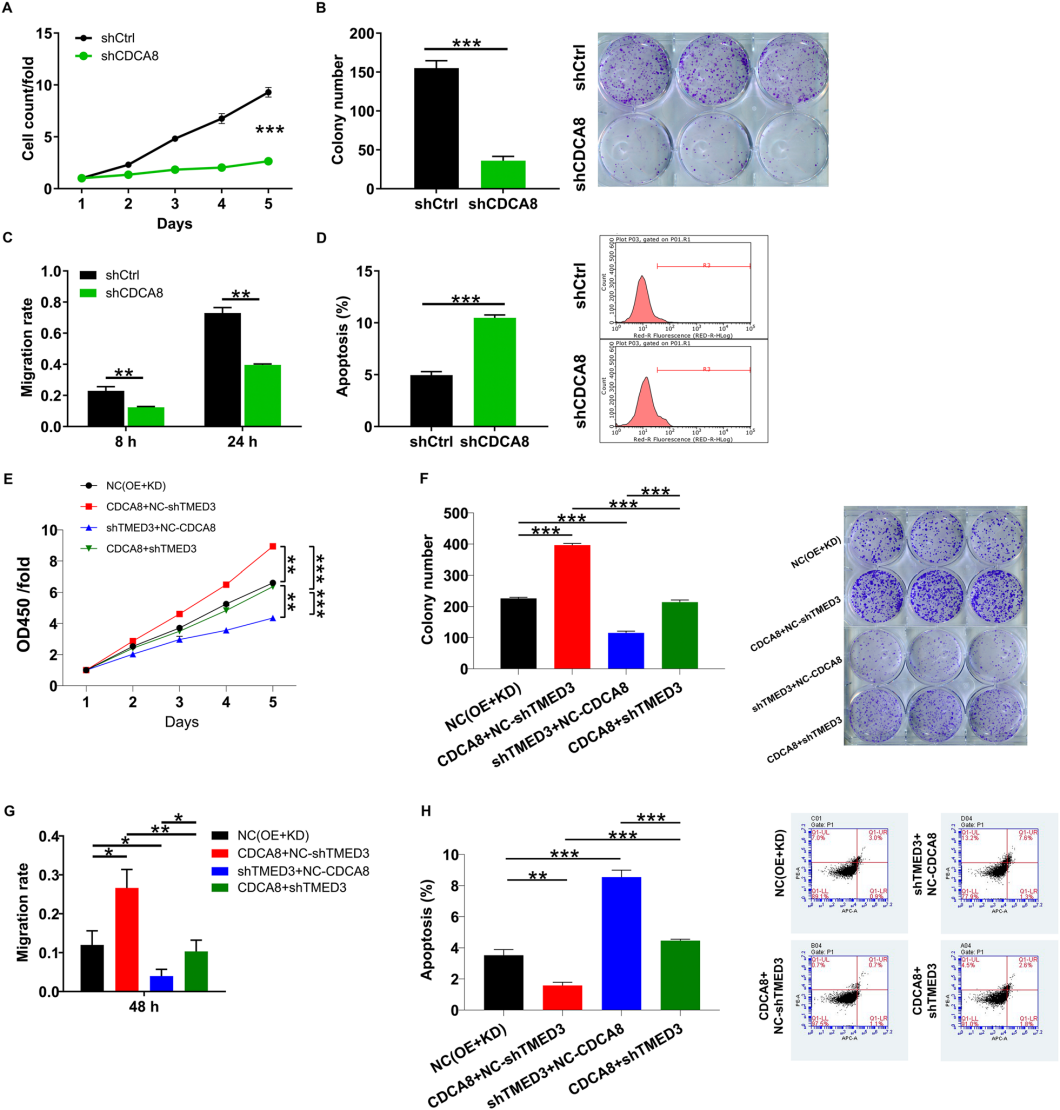
Elevating CDCA8 abolished the suppression of cell proliferation and migration from TMED3 depletion. **A** Celigo cell counting assay was performed to examine the effect of CDCA8 downregulation on A375 cell proliferation. **B** The ability of CDCA8-depleted A375 cells to form colonies was assessed. **C** The effect of CDCA8 downregulation on A375 cell migration was evaluated by wound-healing assay. **D** Flow cytometry was performed to visualize the changes of A375 cell apoptosis upon knocking down CDCA8. **E**–**H** The abilities of cell proliferation (**E**), colony formation (**F**), migration (**G**) and apoptosis (**H**) were assessed via CCK8 assay, colony formation assay, wound-healing experiment and flow cytometry analysis, respectively. NC (OE + KD): Control; CDCA8 + NC-shTMED3: CDCA8 overexpression; shTMED3 + NC-CDCA8: TMED3 downregulation; CDCA8 + shTMED3: CDCA8 overexpression combining with TMED3 downregulation. The data were expressed as mean ± SD. * *P* < 0.05, ** *P* < 0.01, *** *P* < 0.001

Finally, we proposed whether TMED3 modulates MM development by mediating CDCA8. To verify this hypothesis, we developed lentiviral vectors with CDCA8 overexpression, CDCA8 knockdown as well as TMED3 knockdown, which were employed alone or in combination to infect A375 cells (Additional file 1: Fig. S1). We found that silencing CDCA8 blocked cell proliferation, colony formation and migration, while enhanced cell apoptosis (Fig. 5A–D). In contrast, overexpressing CDCA8 acquired a trend opposite to its downregulation (Fig. 5E–H). More importantly, elevating CDCA8 partially rescued the inhibition of TMED3 knockdown on cell proliferation, colony formation and migration, and eliminated the promoting effect on apoptosis (Fig. 5E–H). In summary, we proposed that TMED3 accelerated MM development via targeting CDCA8.

**Fig. 6:**
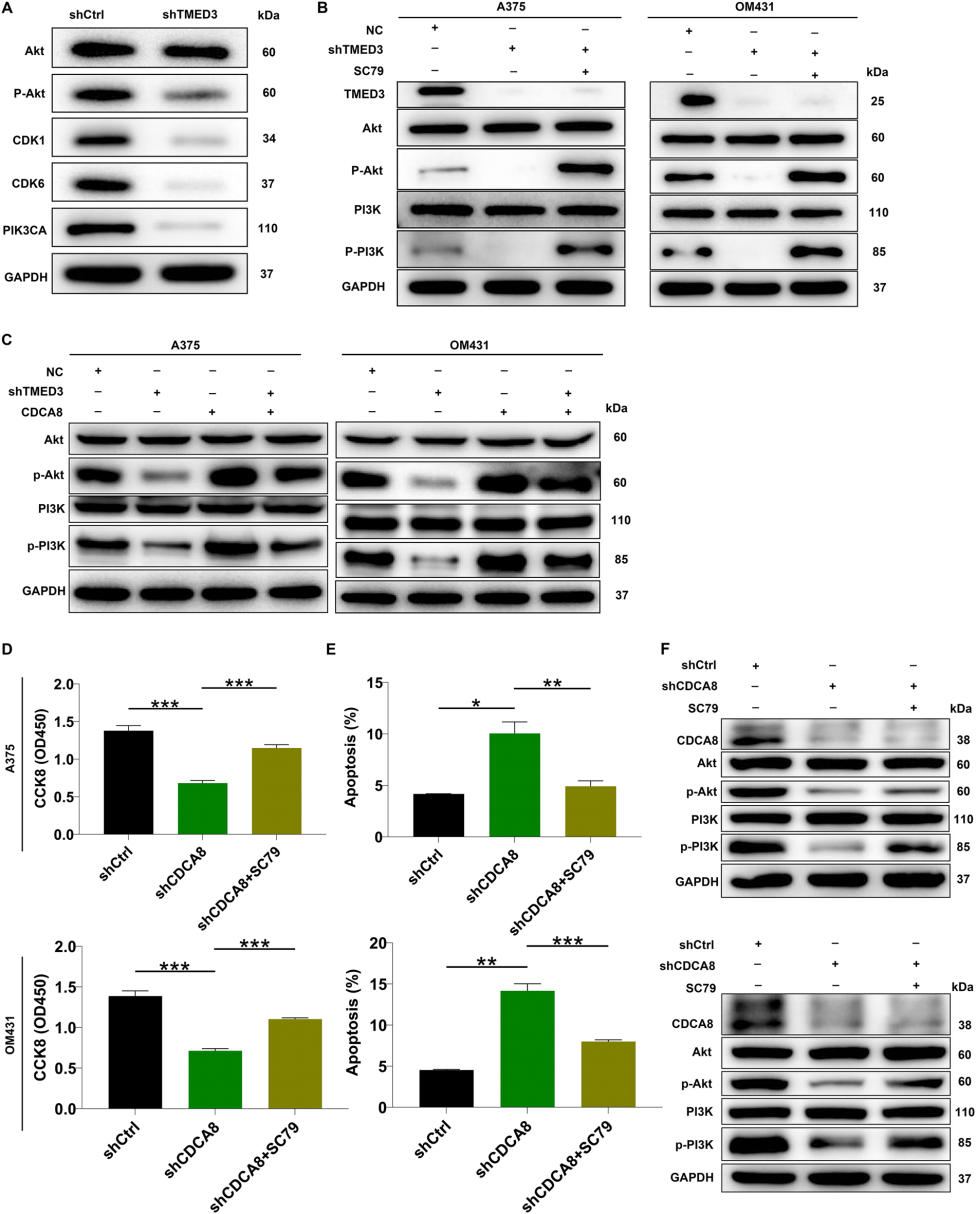
TMED3 mediates melanoma development via moderating PI3K/AKT signaling pathway. **A** Western blotting was employed to reveal the protein expression of AKT, p-AKT, CDK1, CDK6 and PIK3CA in response to TMED3 knockdown in A375 cells. **B** Western blot experiment showed the changes AKT, PI3K and their phosphorylation levels in shTMED3 A375 and OM431 cells after treatment with or without SC79. **C** The levels of AKT, PI3K and their phosphorylation levels in A375 and OM431 cells with CDCA8 overexpression and/or TMED3 downregulation. **D**, **E** Cell proliferation (**D**) and apoptosis (**E**) in CDCA8-depleted A375 and OM431 cells were analyzed following SC79 addition. **F** The alterations in CDCA8, Akt, PI3K, p-Akt and p-PI3K expression in CDCA8-depleted A375 and OM431 cells following SC79 addition. The data were expressed as mean ± SD. * *P* < 0.05, ** *P* < 0.01, *** *P* < 0.001

On the other hand, a question how TMED3/CDCA8 induces MM development arose. Thus, several common cancer-associated elements were monitored, demonstrating that the patterns of p-AKT, CDK1, CDK6 and PIK3CA were reduced in response to TMED3 depletion (Fig. 6A). In addition, the above-mentioned network relationship revealed that TMED3 was related to PI3K/AKT signaling pathway. Here, we surmised that PI3K-Akt signaling pathway was a possible target by which TMED3/CDCA8 promotes MM cell proliferation and migration. We thus treated TMED3-depleted A375 and OM431 cell models using AKT activator SC79, showing that the phosphorylation levels of Akt and PI3K were attenuated in TMED3-deficient cells, whereas their expression was raised upon SC79 treatment (Fig. 6B). Given that CDCA8 is a direct target of TMED3, it would be interesting to know whether TMED3 regulates the PI3K-AKT pathway via CDCA8. Consequently, we analyzed the total protein and phosphorylation levels of Akt and PI3K in TMED3-deficient cells after elevating CDCA8. The results again showed the phosphorylation levels of Akt and PI3K were decreased in response to TMED3 knockdown, however, which was rescued after overexpressing CDCA8, but the total protein and phosphorylation levels of Akt and PI3K were unchanged (Fig. 6C), implying that TMED3 regulates PI3K-AKT pathway via CDCA8. Also, previously impaired cell events due to CDCA8 depletion were ameliorated after SC79 addition (Fig. 6D, E). Furthermore, p-Akt and p-PI3K were increased in CDCA8-silenced cells after SC79 treatment (Fig. 6F). These data suggested that TMED3 regulates PI3K-AKT pathway via CDCA8, thereby promoting MM development.

## Discussion

The clinical significance of TMED3 has been also widely investigated in other cancers. For instance, TMED3 was highly expressed in prostate cancer and metastatic prostate cancer, and high expression of TMED3 was associated with the expression of the androgen receptor and ERG oncogene [17]. Furthermore, TMED3 overexpression was significantly correlated with poor prognosis in patients with hepatocellular carcinoma and clear cell renal cell carcinoma [16, 18]. However, the exact roles of TMED3 in MM have rarely been reported.

The present study explored the potential physiological functions and molecular mechanisms of TMED3 in MM. Findings showed that the expression of TMED3 was increased in MM tissues and cell lines, which was associated with lymphatic metastasis and pathological stage. Additionally, silencing TMED3 suppressed cell proliferation and tumor growth in melanoma cell and animal models. These data identified a tumor-promoting role of TMED3 in MM outgrowth. More importantly, we demonstrated that TMED3 promotes melanoma cell proliferation and migration through a mechanism that targets CDCA8 and involves the PI3K/AKT signaling pathway. From one aspect, TMED3 depletion was accompanied by the downregulation of CDCA8. More interestingly, there was an endogenous and exogenous protein interaction between TMED3 and CDCA8. From this, we inferred that TMED3 promotes the proliferation and migration of melanoma cells via targeting CDCA8.

Cell division cycle associated 8 (CDCA8), also akas Borealin/Dasra B, is a member of the chromosome passenger complex (CPC) and is essential for genome delivery during cell division [19]. During cytokinesis, CPC localizes to the inner centromeres, and then performs a series of biological functions including promoting midzone organization, regulating furrow contractility, and specifying the cleavage plane [20–22]. Therefore, the CDCA proteins play an important role in mitosis, intersecting chromosome segregation and cell division with cancer [23]. Actually, CDCA8 is transcriptionally activated in human embryonic stem cells (hESCs) and cancer cells, but slightly, or even absently, expressed in normal tissues. Previous studies demonstrated that overexpression of CDCA8 was responsible for cancer growth and progression [24]. Here, we found that CDCA8 was abundantly expressed in MM. Elevating/silencing CDCA8 could promote/suppress cell functions involved in MM tumor growth. What’s more noteworthy was that CDCA8 elevation could partially reverse the inhibitory effects of TMED3 knockdown on MM malignant behaviors.

From another aspect, we found that the phosphorylation levels of AKT and PI3K were decreased upon knocking down TMED3. What's more striking was that p-AKT and p-PI3K were augmented in TMED3-deficient cells upon SC79 treatment. From these, we speculated that TMED3 might enhance MM development via moderating PI3K/AKT signaling pathway. It is well-known that PI3K/AKT signaling pathway is one of the most important signaling pathways involved in normal cellular processes. Its aberrant activation modulates autophagy, epithelial-mesenchymal transition, apoptosis, chemoresistance, and metastasis in many human cancers [25]. More importantly, PI3K-Akt signaling pathway is a potential target by which multiple genes promotes many types of cancer cell proliferation and migration [26–28]. To further evidence that TMED3 regulated the PI3K-AKT pathway via CDCA8, we detected the levels of Akt and PI3K in TMED3-silenced cells with CDCA8 overexpression, and found an increase in p-Akt and p-PI3K and no obvious alterations in Akt and PI3K. Additionally, previous studies have reported the link between CDCA8 and PI3K/AKT signaling pathway. For example, CDCA8 was identified as a key gene in osteosarcoma, it and other hub genes were mainly enriched in PI3K-Akt signaling pathway [29]. Another study demonstrated that that CDCA8-regulated gene sets associated with PI3K/AKT/mTOR signaling [30]. Here, cell function experiments demonstrated that CDCA8 depletion restrained proliferation and accelerated apoptosis, while these behaviors were halted following the addition of SC79. At the molecular level, p-Akt and p-PI3K were increased in CDCA8-silenced cells after SC79 treatment. These outcomes implied that TMED3 regulated PI3K-AKT pathway via CDCA8, thereby promoting MM development.

## Conclusions

In conclusion, our study identified TMED3 as a tumor promoter in MM, which was upregulated in MM, thereby promoting MM by regulating cell proliferation, apoptosis and cell migration. Therefore, TMED3 might be considered as a novel therapeutic target, and its knockdown could serve as a promising MM therapeutic strategy.

## Methods

### Tissue specimens and cell lines

A tissue microarray containing 167 melanoma tissues and 30 normal tissues were provided by Xi’an Alenabio Co., Ltd. (Xi’an, China). All patients signed informed consent forms. Studies involving human participants were reviewed and approved by Ethics Committee of Shanghai Tenth People’s Hospital.

Five human melanoma cell lines used here, A375, SK-MEL-28, MUM-2B, MUM-2C and OM431, were purchased from Cell Resource Center, Institute of Basic Medicine, Chinese Academy of Medical Sciences (Beijing, China). MUM-2B and MUM-2C cells were grown in H-DMEM medium + 10% FBS. A375 cells were cultured in DMEM medium + 10% FBS. SK-MEL-28 and OM431cells were cultured in 1640 medium + 10% FBS. All cells were incubated in a 37 °C incubator with 5% CO_2_.

### Immunohistochemistry (IHC) staining

First, the tissue slides were put in the oven at 65 °C for 30 min. Then, the slides were put into xylene for dewaxing, which were subsequently washed using alcohol (China National Pharmaceutical Group Co., Ltd, Beijing, China). Next, the slides were repaired with 1 × EDTA (Beyotime Biotechnology Co., Ltd, Shanghai, China) and blocked with 3% H_2_O_2_ and serum. After that, the slides were incubated with primary antibodies and secondary antibodies at 4 °C overnight, and then were stained with DAB and hematoxylin (Baso DiagnosticsInc., Zhuhai, China). Finally, the slides were sealed with neutral resin (China National Pharmaceutical Group Co., Ltd, Beijing, China). The images were scored as negative (0), positive [1–4], +  + positive [5–8], or +  +  + positive [9–12], with reference to the sum of the staining intensity (varied from weak to strong) and staining extent scores, which graded as 0 (0%), 1 (1–25%), 2 (26–50%), 3 (51–75%), or 4 (76–100%). Antibodies were listed in Additional file 1: Table S1.

### Plasmid construction and lentivirus infection

The TMED3 and CDCA8 shRNA target sequences, as well as CDCA8 overexpression plasmids were designed by Shanghai Bioscienceres Co., Ltd. (Shanghai, China), and subsequently were inserted into the BR-V-108 vector through the restriction sites at both ends and transformed into TOP 10 E. coli competent cells (Tiangen, Beijing, China). The plasmids of positive recombinants were extracted with the EndoFree maxi plasmid kit (Tiangen, Beijing, China), and the concentration of which was determined by a spectrophotometer (Thermo_Nanodrop 2000). A375 and OM431 cells were infected via adding 20 μL 1 × 10^8^ TU/mL lentivirus, and then were respectively cultured in DMEM medium and 1640 medium (both containing 10% FBS) in a 6-well dish (2 × 10^5^ cells/well). Finally, the infection efficiencies and knockdown/overexpression efficiencies were evaluated by microscopic fluorescence, qRT-PCR and western blot.

### RNA extraction, cDNA synthesis and qRT-PCR

After infection, total RNA of A375 and OM431 cells was extracted using TRIzol reagent (Sigma, St. Louis, MO, USA) for cDNA synthesis. qRT-PCR assay was performed via using the Promega M-MLV Kit (Promega Corporation, Madison, Wisconsin, USA) and the SYBR Green Mastermixs Kit (Vazyme, Nanjing, Jiangsu, China). GAPDH was as an internal normalization control. The relative expression of mRNA was evaluated based on the 2^−△△Ct^ method. The primers sequences (5ʹ-3′) were listed in Additional file 1: Table S2.

### Western blot assay and Co-immunoprecipitation (Co-IP)

A375 and OM431 cells were collected after being infected indicated lentivirus to harvest protein. The protein purity was quantified with BCA methods. After that, total proteins were segregated using 10% SDS-PAGE and then transferred into PVDF membranes. Next, the membranes were subject to be blocked in TBST solution with 5% non-fat milk and subsequently incubated with primary antibodies and secondary antibodies, and then washed with TBST solution for three times (10 min/time). Finally, the ECL + plusTM Western blotting system kit was used for color rendering to capture X-ray imaging.

In terms of exogenous Co-IP assay, the proteins of 293 T cells infected with TMED3-Flag were immunoprecipitated with Flag, CDCA8 or IgG and then subjected to western blot. For endogenous Co-IP analysis, the proteins of A375 and OM431 cells were immunoprecipitated with IgG and anti-TMED3. After that, the proteins in the immunocomplex were separated by 10% SDS-PAGE and used for TMED3 and CDCA8 incubation. Antibodies were showed in Additional file 1: Table S1.

### Cell proliferation detection and colony formation assay

In this study, cell proliferation was assessed by three methods including MTT assay, Celigo cell counting assay and CCK8 experiment. For MTT assay, A375 and OM431 cells with indicated lentivirus were seeded in a 96-well plate (2000/well). 20 μL MTT (5 mg/mL) and 100 μL DMSO were added. OD value at 490 nm wavelength was detected using a microplate reader (Tecan infinite, Mannedorf Zurich, Switzerland) for 5 days. In terms of Celigo cell counting assay, the cell images were taken by Celigo image cytometer (Nexcelom Bioscience, Lawrence, MA, USA), and a continuous 5-day cell proliferation curve was drawn. Another method was CCK8 experiment, A375 and OM431 cells with indicated lentivirus were cultured in a 96-well plate at the density of 2500 cells/well. 10 μL CCK8 reagent was added, and then the 96-well plate was oscillated for 2–5 min. OD value was detected for continuous 5 days by Microplate Reader (Tecan infinite) at 450 nm.

### Colony formation assay

Lentivirus infected cells were seeded in a 6-well plate (1000 cells/well). 5 days later, the cells were washed with PBS, fixed with 1 mL 4% paraformaldehyde and stained by 500 μL Giemsa (Dingguo, Shanghai, China) to calculate the number of colonies.

### Cell migration assay

Cell migration was assessed by transwell assay and wound-healing assay. A375 and OM431 cells infected with indicated lentivirus were seeded in a 24-well plate (1 × 10^5^ cells/well). Subsequently, the cells were loaded into the upper chamber containing serum-free medium. Then, the upper chamber was transferred to the lower chamber with 30% FBS and incubated for 72 h. Finally, 400 µL Giemsa was used for cell staining and the cell migration ability was quantified. For wound-healing assay, cells were cultured in a 6-well plate at a density of 5 × 10^4^ cell/well for 12 h and then wounded to generate a straight scratch. After cells were washed with PBS gently, serum-free medium was used to culture cells at 37 °C. Pictures were taken by a light microscope (DFC500; Leica) at appropriate time after wounding.

### Flow cytometry assay

The abilities of cell apoptosis were assessed by flow cytometry using the Annexin V-APC single-staining or Annexin V-APC/PI double-staining method. In detail, lentivirus-infected A375 and OM431 cells were seeded in a 6-well plate at a density of 2 mL/well and cultured for 5 days. Then, 10 μL Annexin V-APC was added for staining at room temperature in the dark. The cell apoptosis level was measured by using FACSCalibur (BD Biosciences, San Jose, CA, USA). For double-staining experiment, cells were treated as described above. Differently, 5 μL Annexin V-APC and 5 μL PI solution were successively added before cells were performed on FACSCalibur detection.

### Human apoptosis antibody array

Human Apoptosis Antibody Array was employed in A375 cells to visualize the changes of apoptosis-related protein expression in response to TMED3 depletion. After the cells were lysed, the Handling Array membranes were blocked in 2 mL 1 × Wash Buffer II and incubated with cell lysates and 1 × Biotin-conjugated Anti-Cytokines overnight at 4 °C. Finally, the signals of membranes were tracked by chemiluminescence imaging system.

### PrimeView human gene expression array

Total RNA was extracted as described previously. The quality and integrity of RNA were determined by Nanodrop 2000 (Thremo Fisher Scientific, Waltham, MA, USA) and Agilent 2100 and Agilent RNA 6000 Nano Kit (Agilent, Santa Clara, CA, USA). Referring to the manufacturer’s instruction, RNA sequencing was performed with Affymetrix human GeneChip PrimeView and the data were scanned by Affymetrix Scanner 3000 (Affymetrix, Santa Clara, CA, USA). The statistical significance of raw data was completed by using a Welch *t*-test with Benjamini–Hochberg FDR (|Fold Change|≥ 1.3 and *FDR* < 0.05 as significant). Significant difference analysis and functional analysis based on Ingenuity Pathway Analysis (IPA) (Qiagen, Hilden, Germany) was executed, and |Z—score|> 0 is considered valuable.

### The cancer genome atlas (TCGA) database analysis

The relationship between TMED3 and CDCA8 was established based on 103 primary SKCM tumor RNAseq samples from the TCGA database. Pearson correlations of gene expression values were calculated using the R cor.test function and plotted using the R packet ggplot2.

### The construction of nude mouse tumor formation model

Female BALB-c nude mice (Four-week-old) were obtained from Shanghai Lingchang Animal Research Co., Ltd., and their care was conducted according to the ARRIVE guidelines. All animal experiments conformed to the European Parliament Directive (2010/63/EU) and were approved by the Ethics Committee of Shanghai Tenth People’s Hospital. The mice were randomly divided into indicated groups (10 mice/group) and were subcutaneously injected 5 × 10^6^ A375 cells with shTMED3 or shCtrl. The tumor volume was tested during the entire feeding period. 0.7% sodium pentobarbital was injected intraperitoneally and the fluorescence was observed by the in vivo imaging system (IVIS Spectrum, Perkin Elmer). 17 days later, the mice were sacrificed and the tumors were removed to weigh and photograph, and finally frozen in liquid nitrogen and stored at − 80 °C.

### Statistical analysis

Statistical analysis was carried out using GraphPad Prism 8 (San Diego, CA, USA) and SPSS 19.0 (IBM, SPSS, Chicago, IL, USA). All data were represented as the mean ± SD from at least three repeated experiments. Student’s t-test (for comparisons of two groups) and one-way ANOVA (for multiple group comparisons) were used to analyze the statistical significance. The Spearman correlation analysis and Mann–Whitney U analysis were used to assess the association between TMED3 expression and pathological characteristics of MM patients. *P* < 0.05 was considered to be statistically significant. All the experiments were in triplicate.

## Supplementary Information

**Additional file 1: Figure S1.** (A) The infection efficiencies of CDCA8, shCDCA8 and shTMED3+CDCA8 in A375 cells were evaluated through observing the fluorescence inside cells. Magnification times: 200×. (B, C) The TMED3 and CDCA8 mRNA (B) and protein (C) expression in A375 cell lines after infecting CDCA8, shCDCA8 and shTMED3+CDCA8 was analyzed by qRT-PCR and western blot. * P < 0.05, ** P < 0.01. **Table S1.** Antibodies used in western blotting and IHC. **Table S2.** Primers used in qRT-PCR.

## Data Availability

The datasets used and/or analysed during the current study are available from the corresponding author on reasonable request.
