# Supplementary material for: TMED3 promotes the development of malignant melanoma by targeting CDCA8 and regulating PI3K/Akt pathway
Source: Cell Biosci. 2023 Mar 29;13:65. doi: 10.1186/s13578-023-01006-6 (PMC10053972; doi:10.1186/s13578-023-01006-6)
Supplement: Supplementary file 1 — Additional file 1: Figure S1. (A) The infection efficiencies of CDCA8, shCDCA8 and shTMED3+CDCA8 in A375 cells were evaluated through observing the fluorescence inside cells. Magnification times: 200×. (B, C) The TMED3 and CDCA8 mRNA (B) and protein (C) expression in A375 cell lines after infecting CDCA8, shCDCA8 and shTMED3+CDCA8 was analyzed by qRT-PCR and western blot. * P < 0.05, ** P < 0.01. Table S1. Antibodies used in western blotting and IHC. Table S2. Primers used in qRT-PCR. [file 13578_2023_1006_MOESM1_ESM.docx]

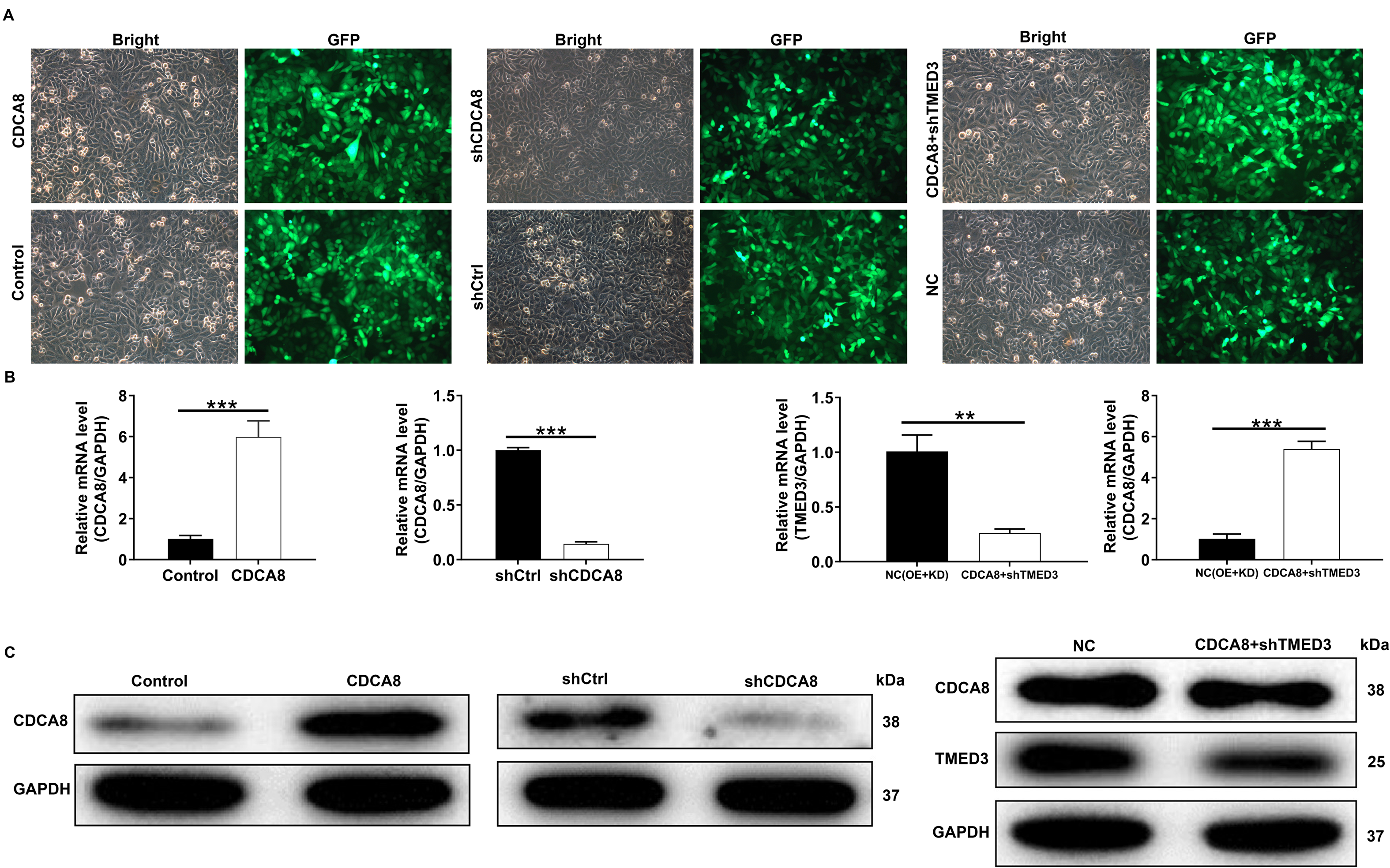


**Figure S1.** (A) The infection efficiencies of CDCA8, shCDCA8 and shTMED3+CDCA8 in A375 cells were evaluated through observing the fluorescence inside cells. Magniﬁcation times: 200×. (B, C) The TMED3 and CDCA8 mRNA (B) and protein (C) expression in A375 cell lines after infecting CDCA8, shCDCA8 and shTMED3+CDCA8 was analyzed by qRT-PCR and western blot. * *P* < 0.05, ** *P* < 0.01.

Additional Table S1. Antibodies used in western blotting and IHC.

| Primary antibodies | Dilution in WB | Source species | Company | Catalog No. |
| --- | --- | --- | --- | --- |
| TMED3 | 1:1000 | Rabbit | abcam | ab223175 |
| Akt | 1:1000 | Rabbit | CST | 4685 |
| P-Akt | 1:500 | Rabbit | R&D | AF887-sp |
| CDK1 | 1:1000 | Rabbit | abcam | ab133327 |
| CDK6 | 1:1000 | Rabbit | abcam | ab151247 |
| PIK3CA | 1:1000 | Rabbit | abcam | ab40776 |
| PI3K | 1:2000 | Mouse | Sanying,Wuhan | 67071-1-Ig |
| p-PI3K | 1:750 | Rabbit | BIOSS | bs-3332R |
| E2F1 | 1:1000 | Rabbit | abcam | ab179445 |
| CDCA8 | 1:1000 | Rabbit | abcam | ab74473 |
| PNO1 | 1:1000 | Rabbit | biorbyt | orb78411 |
| RPL31 | 1:1000 | Rabbit | abcam | ab103991 |
| N-cadherin | 1:1000 | Rabbit | abcam | ab18203 |
| Vimentin | 1:1000 | Rabbit | abcam | ab92547 |
| Snail | 1:1000 | Rabbit | CST | 3879S |
| Flag | 1:50/1:2000 | Mouse | Proteintech | 66008-3-Ig |
| GAPDH | 1:3000 | Rabbit | Bioworld | AP0063 |
| DYKDDDDK Tag | 1:50/1:1000 | Rabbit | CST | 14793 |
| Secondary antibody | Dilution |  | Company | Catalog No. |
| Goat Anti-Rabbit | 1:3000 |  | Beyotime | A0208 |
| Goat Anti- Mouse | 1:3000 |  | Beyotime | A0216 |

| Primary antibodies | Dilution in IHC | Source species | Company | Catalog No. |
| --- | --- | --- | --- | --- |
| TMED3 | 1:20 | Rabbit | abcam | ab151056 |
| CDCA8  Ki67 | 1:30  1:200 | Rabbit  Rabbit | abcam  abcam | ab70910  Ab16667 |
| Secondary antibody | Dilution |  | Company | Catalog No. |
| Goat Anti-Rabbit | 1:400 |  | abcam | A6721 |

Additional Table S2. Primers used in qRT-PCR.

| **Gene** | **Forward primer sequence (5′-3′)** | **Reverse primer sequence (5′-3′)** |
| --- | --- | --- |
| TMED3 | GGCGTGAAGTTCTCCCTGGATT | GCTGTCGTACTGCTTCTTCGTTTC |
| SLC27A1 | CTGGACCCGCTGCTGAAGGA | CCCCGACGTGTAGATGTAGAAAAGA |
| ABL1 | TTCTGATGGCAAGCTCTACGT | TTTGGGGCTGGATAATGGA |
| ADIPOR1 | TGTGCTCCCTGACTGGCTAA | CTGTATGAATGCGGAAGATGCT |
| PHGDH | GGACAATGTGGATCTGGAGGCC | TCTGCGGCACTGAGGCTGTT |
| BRCA1 | TGGCAACATACCATCTTCAACC | TGTCAATTCTGGCTTCTCCCT |
| FEN1 | GCAGAATGAGGAGGGTGAGACCA | GGCTTGCCATCAAAGACATACACG |
| POLA1 | CCGTGTCCTACTTAGGAAGTTTTC | CCTCATCTGCCCCTTTTACC |
| E2F1 | CACTTTCGGCCCTTTTGCTC | GTGCTCTCACCGTCCTACAC |
| KIF3B | AAAATCCTGGAGCAGAAACG | TTGGTCTTGATGTCCACCTCTT |
| FANCD2 | GGCTTGACAGAGTTGTGGATG | TAGGATCTCAGGTAGGCTGGTG |
| CENPN | CAGTGATGCTGCCCTGTTAGA | CATCTTCACCTGGTCCTTTACTCA |
| MAPK3 | ATTGTGCAGGACCTGATGGA | ACGTTGGCGGAGTGGATGTA |
| KPNA1 | TCTACCCACAGGCAACTTTGAA | GCGAGAAAGAAAAACCCCTTGA |
| MCM10 | TCAGTACCATGTCCAGGCTCA | CTCTGCGGGCAAACTTCTT |
| SKP2 | ATAGAAGTGTCCACCCTCCACG | CACCCAGAAAGGTTAAGTCGC |
| CDCA8 | TTGAGTCAGACAGGCAGAACC | TTCCTCCAAGGGCGAAGTAG |
| PIK3CD | GTGAACGGCAGGCATGAGTA | AGGATGGAGGAGGAATGGAC |
| PNO1 | GGAACATTTGGGACTTCAGATACG | CCACCTGAAAGCCGAGAATAAA |
| PRKAR1B | TGACCAACATCAGCGAGGGA | AGCGTGCTGCCCATAAGGAT |
| RPL31 | GCTCAACAAAGCTGTCTGGG | TTATGGGCTCTTGGCGACT |
| GAPDH | TGACTTCAACAGCGACACCCA | CACCCTGTTGCTGTAGCCAAA |
